# Morphological, Biochemical, and Molecular Diversity of an Indian Ex Situ Collection of Pomegranate (*Punica granatum* L.)

**DOI:** 10.3390/plants11243518

**Published:** 2022-12-14

**Authors:** Shilpa Parashuram, Nripendra Vikram Singh, Nilesh Nivrutti Gaikwad, Giandomenico Corrado, P. Roopa Sowjanya, Boris Basile, Nitesh Shirur Devaraja, Ram Chandra, Karuppannan Dhinesh Babu, Prakash Goudappa Patil, Pradeep Kumar, Akath Singh, Rajiv Arvind Marathe

**Affiliations:** 1 ICAR-National Research Centre on Pomegranate, Kegaon, Solpaur 413255, Maharashtra, India; 2 Department of Agricultural Sciences, University of Naples Federico II, 80138 Portici, NA, Italy; 3 Department of Genetics and Plant Breeding, Chandra Shekhar Azad University of Agriculture & Technology, Kanpur 208002, Utter Pradesh, India; 4 ICAR-Central Arid Zone Research Institute, Jodhpur 342003, Rajasthan, India

**Keywords:** fruit trees, germplasm, plant genetic resource, diversity, morphological traits, biochemical parameters, DNA, Simple Sequence Repeats

## Abstract

Pomegranate (*Punica granatum*, L.) is a fruit tree that is increasingly popular worldwide due to the health-related properties of the fruit juice. While several studies highlighted the rich phytochemical diversity, few efforts have been devoted to an integrative understanding of the level of diversity of this species. This study investigated the diversity of 40 pomegranate accessions in an Indian ex situ collection by using twenty-nine morphological traits, six biochemical parameters, and twenty-nine Simple Sequence Repeats (SSR) markers. Among the evaluated traits, fruit volume (23.34% CV), fruit weight (21.12% CV), and fruit color (*a) (22.69 % CV) largely contributed to the morphological classification. Based on Mahalanobis D^2^ distance and Tocher’s clustering, the 40 pomegranate accessions were grouped into eight clusters, partly consistent with their origin. Specifically, cultivars introduced from foreign countries were present in distinct clusters. The SSR marker analysis generated 66 alleles. The observed heterozygosity values ranged from 0.05 to 0.63, with a mean value of 0.30. Maximum molecular genetic dissimilarity was observed between ‘IC-318720′ and ‘Gul-e-Shah Red’ (0.30). The neighbor-joining dendrogram separated wild accessions from cultivated varieties. The combination of morphological, biochemical, and molecular characterization allowed for comprehensively characterizing the pomegranate diversity and provided information on the relationships between the different aspects of the diversity. This work also suggests that the origin of the accessions is an important factor of discrimination and that the level of admixture between local and foreign material is currently limited.

## 1. Introduction

Pomegranate (*Punica granatum* L.) is a deciduous fruit tree typically grown in sub-tropical and tropical regions of the world [1]. The growing awareness of the health benefits of the fruit and its numerous food derivatives has recently widened the global demand for this species [2,3]. Pomegranate is also appreciated in agriculture for its environmental adaptability, such as tolerance to drought and suboptimal edaphic conditions [1]. 

India is currently the world’s leading country in terms of acreage and production [4]. However, while India contributes to about 45% of the global pomegranate production, export is small, only about 2–2.5% of its total yield. In addition to the known limited shelf-life of the fruit, other factors that explain this trend are related to the need to match international quality standards in terms of fruit size, shape, color, and soundness (e.g., damage due to biotic stress and physiological disorders such as sunburns and fruit cracks). 

Varietal diversification is an important policy not only to enhance the productivity and adaptive capacity of the sector but also to increase the international attractiveness of Indian pomegranate production. For instance, while sweetness and low acidity are two desirable features of the typical Indian production, this is also characterized by small-to-medium fruits with a brighter reddish color and a thin rind [5]. Moreover, it should not be overlooked that most of the Indian fruits come from a very limited number of varieties (i.e., Bhagawa, Phule Arakta, Mridula, Ganesh, and Ruby) [6]. The narrow genetic base of the Indian cropping system represents an inherent risk in terms of resistance to stresses and adaptability to climate change [7]. In addition, current market dynamics indicate that advances in the manufacturing the raw material (e.g., aril and juice extraction) are supporting the expansion of pomegranate worldwide [8,9], driven by the rising consumer demand for strongly colored fruit juices rich in functional ingredients [10]. The process to obtain syrup, nectar, jelly, or concentrate can also use fruits of lower commercial grades, and it represents a lucrative option to add value to the market chain. In order to satisfy all these specific issues, pomegranate breeding programs need to focus on varieties with a range of desirable characteristics. Therefore, germplasm evaluation should not only be based on yield and aesthetic appeal but also on the health-promoting features of the fruits. 

India has a considerable pomegranate diversity in the form of wild (Western Himalaya) and evergreen cultivars [5,11]. The National Research Centre on Pomegranate (NRCP; Solapur, Maharashtra, India) of the Indian Council of Agricultural Research (ICAR) holds arguably one of the largest collections, made of more than 300 accessions [12]. Characterization of the diversity within the available germplasm is an essential prerequisite for pomegranate breeding. For instance, the ICAR-NRCP and other research institutes, including State Agricultural Universities, have released more than 25 cultivars in the last 50 years, including the two highly popular Bhagawa and Ganesh varieties [13]. In India, previous studies have characterized the pomegranate diversity according to the morphological, physico-chemical, and molecular traits, and their combinations [14,15,16,17,18,19]. Nonetheless, also considering current research in other countries [20], a limited number of reports are available on the characterization of pomegranate germplasm combining morphological (including pomological), biochemical, and molecular analyses. The aim of this work was to perform an integrative characterization of an Indian collection of pomegranates that included feral forms, cultivated accession, and national and foreign cultivars. The molecular identification and the morpho-biochemical characterization of plant germplasm are crucial to understanding the role of the observed diversity and its interactions with agronomically useful traits and, ultimately, to develop effective breeding programs and biotechnological interventions.

## 2. Results and Discussion

### 2.1. Morphological Characterization 

The morphological characterization of the pomegranate germplasm was carried out using 35 quantitatively scored traits. Mean values for each accession and their statistical analysis are presented in Table S1. The accessions showed a statistically significant (ANOVA; *p* < 0.05) variability for all the characters analyzed. Descriptive values of the traits in the germplasm collection are presented in Table 1. 

The coefficient of variation (CV) ranged from 3.07% for Total Soluble Solids to 23.34% (fruit volume), with three traits with a CV value higher than 20% and ten traits with a CV between 10 and 20%. The highest CVs were recorded in traits related to fruits, while usually, the lowest values were noted for leaves (except petiole length and width), flowers and seeds. Overall, it is worth paying attention to the high level of variation for traits that have clear commercial importance, such as fruit weight (21.12%), volume (23.34%), and acidity (18.66%), as well as the number of arils per fruits (19.41%) [5]. Specifically, there were three accessions with a fruit size of <100 g, seven accessions within the 100–200 g range, 18 accessions within 200–300 g, nine accessions within 300–400 g, and three accessions with fruits heavier than 400 g. All the commercial and cultivated varieties of India had an average fruit size higher than 250 g. Although fruit size in pomegranate, and in many fruit trees, can be modulated by thinning [21], pomegranates between 200 and 300 g are generally classified within the small-to-medium range in international markets (class 4 in a 1 to 5 scale) (CODEX STAN 310-2013; www.fao.org, accessed on 1 October 2022) [5] Among the wild genotypes, the collections from Uttarakhand and Jammu, Jammu and Kashmir had a smaller fruit weight (lower than 100 g), compared to the feral accessions from Himachal Pradesh. A medium variation was observed for the rind thickness (14.03%), another parameter of commercial importance. Rind thickness influences water loss, fruit shelf, and suitability for packaging and transport [22,23], and the average value and variation in our germplasm is comparable to or higher than those in other works [24,25]. All the wild accessions could be considered to have a high acidity (>1.5%), but they also had a comparable TSS content to the cultivated accessions. The TSS ranged in the whole collection from 15.09 to 19.34 °Brix. This parameter is strongly related to all the sugar forms in the fruit [26], and further analysis is necessary to evaluate differences in the simple carbohydrates influencing taste [27]. Among the variables related to the color space of the fruits and arils, it is interesting that the color coordinate a* (which expresses variation on the green–red axis) was the most variable (CV of 22.69% and 19.55% for the fruits and arils, respectively), consistent with the predominant role of this color component in the visual appearance of the commercial products.

The analysis of the quantitative traits was complemented by scoring three qualitative traits, plant habit and the color of the corolla and calyx (Table S2). All traits were polymorphic. Specifically, an upright tree habit was recorded for only the accession of Russian origin (Gul-e-Shah Red and Gul-e-Shah Rose Pink), while a larger variability was observed for the color traits in terms of possible phenotypes scores. Nonetheless, the only variety of Afghan origin (Kabul Yellow) had paler colors. As expected, there was a strong correlation between the color of the calyx and the corolla. 

### 2.2. Correlation among Quantitatively Scored Morphological Traits 

**Figure 1:**
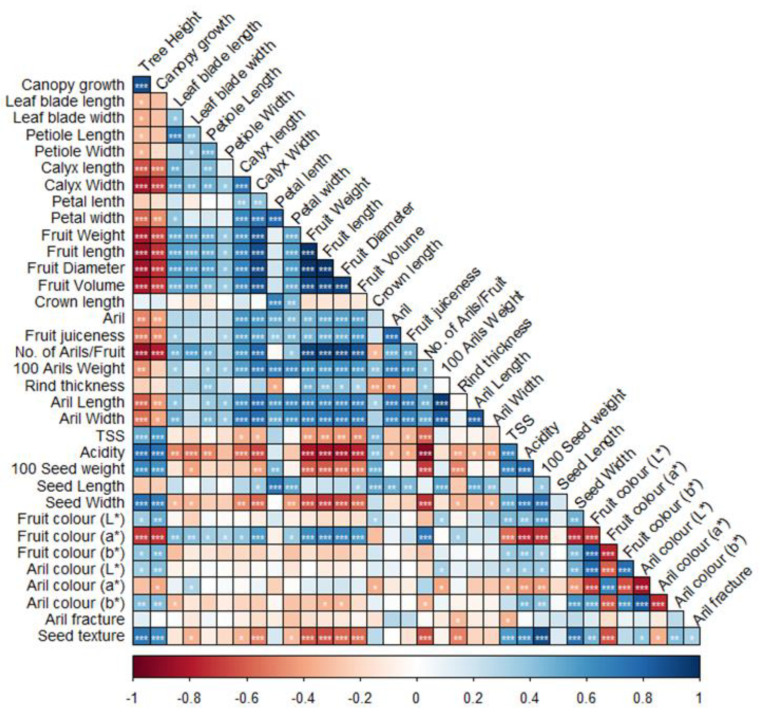
Correlogram of the morphological traits. For each pairwise correlation, the graph displays the correlation coefficient (Pearson) according to the color scale reported at the bottom. Asterisks indicate significant correlation (*: *p* < 0.05; **: *p* < 0.01; ***: *p* < 0.001).

The correlations between the quantitative variables and their significance (*p* < 0.05) are reported in Figure 1. A significant positive correlation was recorded between tree height and canopy growth (r = 0.88); petal length and petal width (r = 0.79); fruit weight and fruit length (r = 0.97); fruit weight and fruit diameter (r = 0.98); fruit weight and fruit volume (r = 0.90); fruit weight and the number of arils/fruit (r = 0.92); fruit volume and number of arils/fruit (r = 0.93); 100 arils weight and aril length (r = 0.94); acidity (%) and TSS (r = 0.66); 100 seed weight and seed texture (r = 0.89); fruit rind color L* and fruit rind color b* value (0.82), and aril color L* value and b* value (r = 0.82). A negative correlation was observed between fruit weight and acidity % (r = −0.80); nunber of arils per fruit and acidity (%) (r = −0.92); acidity (%) and fruit color a* (r = −0.78); fruit rind color a* value and b* value (r = −0.76); aril color L* value and a* value (r = −0.74) (Table S3). Overall, as expected, measurements related to the dimensions of fruits, calyces, petals, or leaves were highly correlated, with the former positively connected with the number and size of the arils. Conversely, tree height and canopy growth were negatively correlated to the dimension of the fruit, calyx, and petals, and with lower value, with the dimension of the leaf blade and petiole.

### 2.3. Non-Hierarchical Clustering of the Accessions Based on the Morphological Traits

The 40 pomegranate accessions were grouped into eight clusters by Tocher’s clustering based on the pairwise Mahalanobis D^2^ distances (Table 2). 

As expected, the largest number of genotypes were grouped in cluster I (14) and cluster II (11) (Table 3). By excluding clusters VII and VIII, made of one accession, the intra-cluster distances, calculated using the Euclidean coefficient, ranged from 51.48 to a maximum of 125.75. Intra-cluster distances were overall consistent with a mutually excluding, progressive hierarchical classification that minimizes the intra-cluster distance and maximizes the inter-cluster distances. The members of clusters 5 and 7 exhibited maximum between-groups divergence (inter-cluster distance of 745.37), followed by clusters 1 and 5 and clusters 2 and 7. The members of clusters 3 and 7 were the least divergent (inter-cluster distance of 154.67). Indian wild accessions were clustered in groups I, III, and VII, and Indian cultivated types were in clusters II, IV, and V. Foreign cultivars introduced from the former USSR (Gul-e-Shah Red and Gul-e-Shah Rose Pink) were grouped in cluster VI, while Kabul Yellow from Afghanistan was the only member of cluster VIII.

The number of clusters and their subdivision imply an important divergence among the accession taking as reference studies based on Tocher’s optimization method in other fruit trees. For instance, 42 mango cultivars were divided into three clusters based on 14 morphological traits [28]. Thirty-six native jabuticaba trees (*Plinia cauliflora*) were divided into four clusters according to 16 fruit traits, with some degree of consistency between clustering and collection sites [29]. The analysis of six features of the latex of 44 genotypes of rubber tree (*Hevea brasiliensis*) indicated a six-cluster division [30].

### 2.4. Analysis of the Biochemical Traits

Pomegranate fruits are highly appreciated for their antioxidant properties and content of bioactive compounds [31]. These are traits of interest in contemporary breeding [32]. For these reasons, we investigated the main classes of bioactive compounds in the juice as well as their antioxidant activity. There were significant (ANOVA; *p* < 0.05) differences among the accession for the four biochemical traits under investigation (Table S4). In particular, the anthocyanins had the highest CV, ranging from a minimum of 0.15 mg/100 mL c3g eq (P-13) to a maximum of 21.75 (Phule Arakta) (Table 4).

It is long known that pomegranate fruits and juices can contain a high level of anthocyanins [33], whose presence is influenced by several factors [34]. The parameter least variable in our population was the total antioxidant activity. TAA was the only biochemical trait that significantly correlated with another, namely the ascorbic acid content (R:0.73; *p* < 0.01; Pearson). This implies that the antioxidant properties of fresh juice are strongly affected by this water-soluble vitamin. Finally, the pomegranate juice had a notable quantity of total polyphenols, reaching a maximum value of 2919.00 mg/mL GA eq for the IC-318793 accession.

### 2.5. Agglomerative Hierarchical Analysis of the Pomegranate Accessions Based on Morphological and Biochemical Data

**Figure 2:**
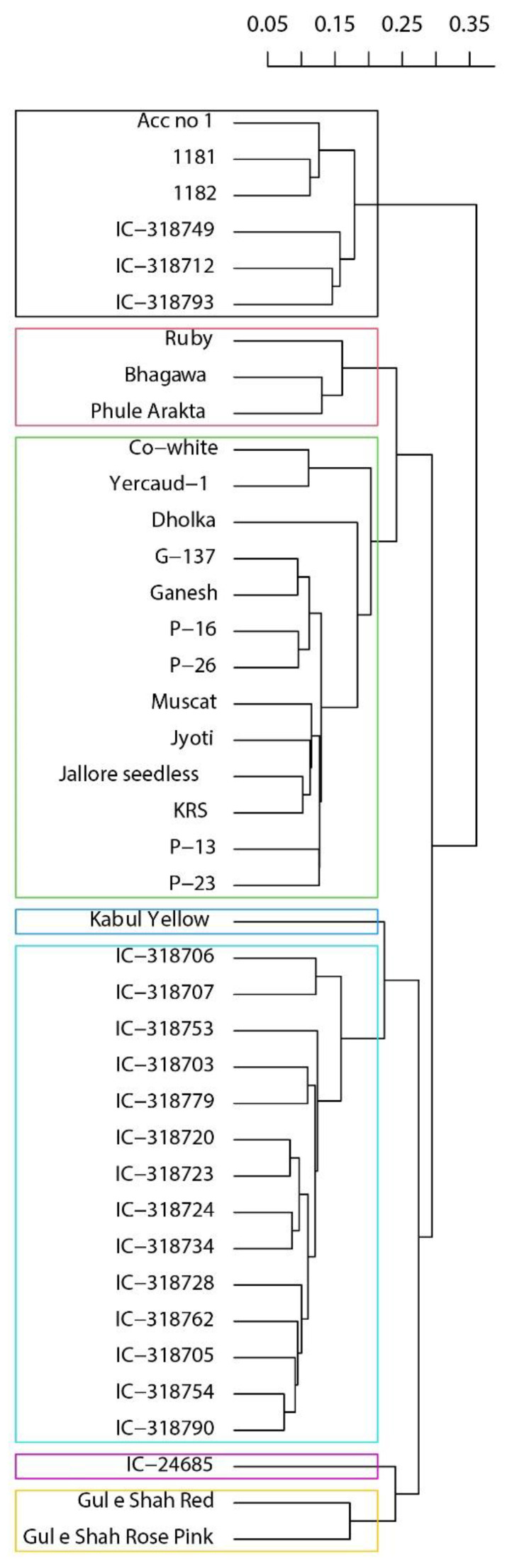
Hierarchical classification of the forty pomegranate accessions using morphological and biochemical data (Gower’s distance; UPGMA algorithm). Rectangles were drawn for k = 7.

In order to classify the accessions considering the whole phenotypic diversity (i.e., the 35 quantitative traits, 3 qualitative traits, and 4 biochemical parameters), we used a hierarchical clustering procedure based on the Gower distance. A sensible biological classification could be depicted at k = 7 (Figure 2). Specifically, at a distance of approximately 0.20, the Afghan and the two Russian cultivars had distinct classifications (blue and yellow rectangles, respectively). Moreover, most of the IC accessions (14 out of 18) are clustered together (azure). While IC-24685, the only accession from Banda (Uttar Pradesh), also had a distinctive position (cyan), the other IC accessions were present in another cluster (black) along with the 1181 and 1182. In addition to this couple, the pairwise similarity was in different instances consistent with the origin of the accessions, as for Phule Arakta-Bhagawa, G-137-Ganesh, P-16-P-26. P13–23, and within the azure cluster, also for the pair IC-318706-IC-318707, IC-318720-IC-318723, and IC-318724-IC-318734.

### 2.6. Molecular Characterization of Pomegranate Accessions 

The SSR technique was employed to evaluate the molecular diversity within the pomegranate accession. These markers have been successfully employed by several researchers to characterize the pomegranate germplasm to study genetic diversity and to understand population structure and association analysis [14,35,36,37].

In total, 29 PGKVR SSR markers were used for genotyping of the selected pomegranate germplasm [38]. They were all polymorphic yielding 66 alleles for an average number of 2.28 alleles per locus (Table 5). Polymorphic information content (PIC) values ranged from 0.05 to 0.56, with an average of 0.25. The PGKVR048, PGKVR045, PGKVR033, and PGKVR093 were the most informative markers according to the PIC value and 0.42, respectively. The observed heterozygosity values ranged from 0.05 to 0.63, with a mean value of 0.30. (Table 5).

**Figure 3:**
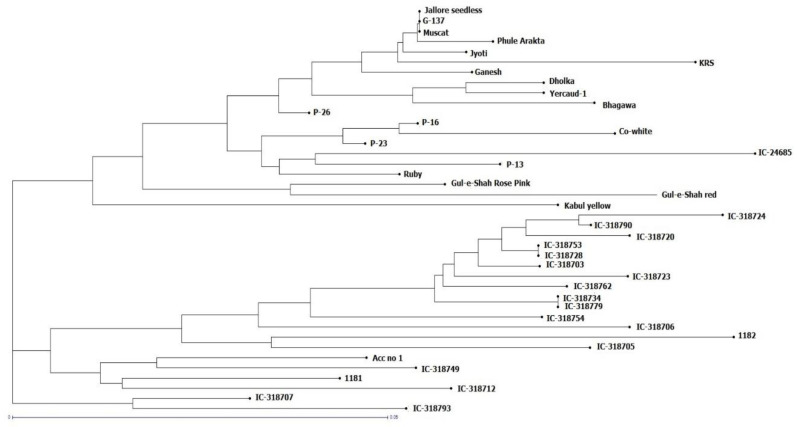
Neighbor-joining clusters of 40 pomegranate accessions generated by SSR markers using the dissimilarity coefficient.

The maximum genetic dissimilarity (0.61 bootstrap value) was observed between ‘IC-318720′ and ‘Gul-e-Shah Red’ (0.30); ‘IC-318724’ and ‘KRS’ (0.29); ‘1182’ and ‘Yercaud-I (0.27); ‘1182’ and ‘Dholka (0.27); ‘1182’ and ‘IC-318712 (0.27); ‘IC-318724’ and ‘Bhagawa (0.27); and IC-24685 and IC-318706 (0.27). Unweighted neighbor-joining cluster analysis divided the accessions into different clusters. All the wild accessions were grouped into clusters I and II, while cultivated varieties were in cluster III. Indian varieties are grouped as sub-cluster-I, foreign varieties (Gul-e-Shah Red and Gul-e-Shah Rose Pink) as sub-cluster-II, and Afghanistan cultivar ‘Kabul yellow’ formed a unique sub-cluster-III under cluster III (Figure 3). 

### 2.7. Relationship between the Clusters Based on Morpho-Biochemical and Molecular Diversity

The resemblance among the 40 accessions depicted by the morpho-biochemical data (Figure 2) and by the molecular markers (Figure 3) was compared in terms of a linear relationship between pairwise distances. The Mantel test indicated a positive (r = 0.53) and significant (*p* < 0.001) correlation between the ordered distance matrices. When taking the result altogether, this implies that the genetically distant accessions have a higher phenotypic diversity than more closely related accessions. In cereals, the association of low marker distances with low phenotypic distances holds true especially for pairs of related lines, and has been related to breeding activities [39,40]. Although quantitative traits can provide a better representation of the resemblance among plant accessions (if compared to qualitative traits) [41], it is also necessary to add that the former are also more sensitive to environmental factors. The comparison of the distances based on morphological and molecular data has yielded a variety of outcomes. Positive correlations are reported, but they are generally lower (r < 0.4). For instance, by limiting the attention to trees, the correlation between AFLP distances and those obtained from quantitative morphological traits was 0.33 in olive [42]. In walnut, the correlation between SSR and morphological data was 0.35 [43]. In apricot, the correlation between SSR and qualitative and qualitative phenotypic data was 0.18 [44]. Finally, the relatively high correlation between molecular and phenotypic distance also gives support to the hypothesis that the observed morphological and biochemical differences have a relevant genetic base with positive implications for breeding.

## 3. Materials and Methods

### 3.1. Experimental Site and Plant Material 

The study was conducted at the Kegaon research farm of the ICAR-National Research Centre on Pomegranate, Solapur, Maharashtra, India (17°68′ N latitude, 75°91′ E longitude; 483.5 m above sea level). The experiment was carried out for two consecutive years during *mrig bahar* (June–July to December–January; 2016–17 and 2017–18). The open-field collection includes cutting-propagated accessions of similar age obtained from various sources (Table 6). Plants management followed standard practices for commercial fruit production, with yearly pruning after fruit harvesting and uniform cultural operations (e.g., weeding, irrigation, fertilization). Major climatic parameters were: 18.57 °C minimum temperature; 32.48 °C minimum temperature; 65.19% min and 90.86% max relative humidity; average annual rainfall: 684.3 mm.

A total of 40 pomegranate accessions were selected for this study (Table 6). They included: five commercial Indian cultivars, which are cultivated over large areas (Bhagawa, Phule Arakta, Ganesh, Ruby); eleven local varieties, which are popular among the native regions of the country (Co-White, Dholka, G-137, Jallore Seedless, Jyoti, KRS, P-13, P-16, P-23, P-26, Yercaud-1); four foreign varieties (Gul-e-Shah Red; Gul-e-Shah Rose Pink; Kabul Yellow; Muscat); one indigenous collection (IC-24685); and twenty feral accessions collected from natural habitats and diversity hotspot regions of the country (1181; 1182; Acc no 1; IC-318703; IC-318705; IC-318706; IC-318707; IC-318712; IC-318720; IC-318723; IC-318724; IC-318728; IC-318734; IC-318749; IC-318753; IC-318754; IC-318762; IC-318779; IC-318790; IC-318793) (Table 6). The trees were spaced 4.5 m × 4 m.

For the morphological characterization, plant material was randomly sampled from around the canopy of three plants per accession, for a total of 15 hermaphrodite flowers at full bloom, 15 adult leaves (from the middle part of the one-year-old shoot), and 30 fruits at maturity. All seeds were extracted from each fruit, counted, and weighed. Arils were hand removed from a total of fifteen randomly selected fruits per accession. Tree measurements (height and canopy growth) were measured with a measuring tape. Linear dimensions of leaves, fruits, seeds, and arils were determined with a digital vernier caliper. Weight was measured using a precision electronic balance. Volumes were determined by the liquid displacement method. The total soluble solids (°Brix) of the juice were determined using a digital refractometer (SMART 1, Atago Co. Ltd., Fukaya, Japan) and expressed in °Brix. The titratable acidity of arils juice was measured by titration using a 0.1 N NaOH solution. Fruit and aril color were examined by using a colorimeter (Lab Scan XE, Hunterlab, VA, USA). The color was expressed using the CIELAB coordinates. L*, a*, and b* (L* is the lightness, with the lowest value 0 yielding black; a* is relative to an axis whose negative values indicate green and positive values red; b* is relative to an axis whose negative values indicate blue and positive values yellow). The texture of the seeds was measured using a texture analyzer (TA-XT Plus, Stable Micro Systems, Godalming, UK). Firmness was expressed as the maximum compression force (N) required to rupture the arils [45]. Aril fracture/bioyield was measured using a texture analyzer (Stable Micro Systems, Godalming, UK).

The qualitatively scored traits were the tree growth habit, the calyx color, and the corolla color, and they were measured on three plants per variety. Plant growth habit was classified as upright, spreading, or weeping. The calyx color was observed when the sepals were closed, considering the following classes: orange, red, and dark red. The corolla color was observed when the flowers were fully open, considering the following classes: white, pink, orange, and red.

### 3.2. Biochemical Analysis

Measurements were performed at the Post-Harvest Technology Laboratory, ICAR-NRC. The juice was extracted from arils with a metal aluminum hand-press juicer (Jasper). All chemicals were purchased from HiMedia (Mumbai, Maharashtra, India). The spectrophotometric analysis was carried out with a GENESYS 50 dual-beam UV-Vis spectrophotometer (Thermo-Fisher Scientific, Greater Mumbai, Mumbai, India).

#### 3.2.1. Total Phenol Content 

Total phenolic compounds were determined by the Folin–Ciocalteu colorimetric method, which is based on the chemical reduction in a mixture of tungsten and molybdenum oxides [46], reading samples at 765 nm [47]. A standard calibration curve of gallic acid was prepared, and the total phenolic content was expressed as mg/L gallic acid equivalents (GAE).

#### 3.2.2. Anthocyanin Content

The content of monomeric anthocyanins (ANT) was estimated with a differential method using two buffer systems: a 25 mM potassium chloride buffer (pH 1.0) and a 0.4 M sodium acetate buffer (pH 4.5) [48]. The absorbance (A) of the samples in each buffer, equilibrated for 1 h, was measured at 510 and 700 nm and calculated according to the following equation: A = (A_510_ − A_700_)_pH1_ − (A_510_ − A_700_)_pH4.5_. The results were expressed as cyanidin-3-glucoside (c3g) equivalents using a molar absorptive coefficient (ε) of 26,900 L/mol·cm, the molecular weight (MW) of 449.2 g/mol, the dilution factor (DF), and the cuvette path length (l, 1 cm), according to the following equation, ANT = (A × MW × DF × 100)/(ε × 1), and it is expressed in mg/100 mL c3g.

#### 3.2.3. Total Antioxidant Activity

The total antioxidant activity (TAA) was determined using a ferric-reducing ability of plasma (FRAP) assay, as previously described [49]. Briefly, a juice sample (150 μL) was added to 4.5 mL of freshly prepared FRAP reagent. The sample was incubated at room temperature for 30 min, and then the absorbance was read at 593 nm against the blank. A standard curve was built using ascorbic acid in the 20–100 μg/mL range. The results were expressed as mg of ascorbic acid equivalent antioxidant capacity per 100 mL of juice.

#### 3.2.4. Ascorbic Acid Content 

Ascorbic acid in the fruit juice was determined using a previously described method based on the reduction in 2,6 di-chlorophenol indophenol by L-ascorbic acid, and it was expressed in mg of ascorbic acid in 100 mL of material [50,51].

### 3.3. DNA Isolation and Molecular Characterization

Genomic DNA was isolated from young leaves following a modified CTAB (Cetyl trimethylammonium bromide) method [52]. The purity and quantity of genomic DNA were determined by using known concentrations of uncut lambda DNA by electrophoresis. Samples were diluted to a 20 ng/μL DNA concentration for polymerase chain reactions (PCR). Twenty-nine SSR primers belonging to the PGKVR series [38] were used for polymorphism detection on the samples. Primers were purchased from (Eurofins, India), and their sequence and main features are reported in Table S5. PCR amplification was carried out in 10 μL reaction volume using the 2X PCR TaqMixture (HiMedia, Mumbai, India); 0.7 μL each of forward and reverse primers (10 pmol), 3.6 μL distilled water, and 20 ng of template DNA. SSR amplification reaction was performed using a Prime-96 thermolcycler (HiMedia, Mumbai, India) with the following thermal profile: 94 °C for 5 min followed by 25 touchdown cycles of 30 s at 94 °C, 30 s at 60 °C (−0.3 °C per cycle), and 45 s at 72 °C, and 15 cycles of 30 s at 94 °C, 30 s at 55 °C, and 45 s at 72 °C, for denaturation, annealing, and primer extension, and final extension time of 7 min at 72 °C [46]. The PCR products were separated through electrophoresis and photographed in a gel documentation system (Vilbert Lourmet, Collégien, France). A standard 100 bp DNA ladder (HiMedia, Mumbai, India) was used to estimate the molecular weight of the amplified products.

### 3.4. Statistical Analysis

Quantitative data were analyzed with a one-way analysis of variance (ANOVA) followed by the Tukey *post hoc* for mean separation (*p* < 0.05). Bivariate correlation analysis was performed using the Pearson coefficient. For the non-hierarchical grouping of the accession based on the morphological traits, we used the Mahalanobis generalized distance (D^2^) and the Tocher’s method [53]. For hierarchical classification, morphological (quantitatively and qualitatively scored traits) and biochemical characteristics were used to compute the pairwise Gower’s distance between accessions [54]. A dendrogram was built using the unweighted pair group method with an arithmetic mean (UPGMA) algorithm. In order to test the correlations between genetic distance matrices and between the morphological and genetic distance matrices among accessions, we used the Mantel test with 10,000 permutations to test for significant correlations (*p* < 0.05). All these analyses were performed in R version 4.2.

For the molecular analysis, each SSR amplification was analyzed by scoring the presence (1) or absence (0) of polymorphic bands in individual lanes. The DARwin 6.0 software [55] was used to estimate genetic dissimilarities. The dissimilarity matrix was analyzed by the unweighted neighbor-joining clustering algorithm. The polymorphic information content (PIC) and heterozygosity of polymorphic SSRs were calculated according to previously published formulas [56].

## 4. Conclusions

The study highlighted the significant differences among the accessions and made evident the pomological characters influencing the classification of the analyzed Indian collection. Moreover, the morphological and molecular marker systems detected a variability that was adequate for the discrimination of all accession. An implication of our work is that the health-promoting activity and the nutraceutical benefits of fruits and juice should always be framed in a specific genotypic context, while pre-clinical studies do not always provide clear indications of the variety employed [8]. Moreover, our result hints towards some country-specific differences that should be further investigated by transnational consortia. The information gained from this study will expand our understanding of the degree of diversity found in Indian ex situ collections of pomegranates and will improve the efficiency of its management, conservation, and utilization. Finally, our work indicates that the collection has the potential to be further evaluated as a source of diversity in breeding programs.

## Figures and Tables

**Table 1:** Descriptive statistical analysis of quantitative traits used for characterization of pomegranate accessions. SD: standard deviation; CV: coefficient of variation.

Character (Unit)	Mean	Min	Max	SD	CV (%)
Tree height (m)	3.14	2.17	4.2	0.48	8.18
Canopy growth (m)	3.49	2.17	4.62	0.61	9.51
Leaf blade length (cm)	5.29	4.2	5.99	0.44	7.56
Leaf blade width (cm)	1.51	1.34	1.74	0.10	7.84
Petiole length (mm)	4.32	3.54	5.21	0.39	11.48
Petiole width (mm)	0.91	0.63	1.22	0.12	17.60
Calyx length (mm)	35.22	27.43	43.72	3.82	8.90
Calyx width (mm)	12.18	9.03	14.25	1.42	8.59
Petal length (mm)	21.06	14.82	25.1	2.14	7.44
Petal width (mm)	15.60	10.55	18.33	1.72	9.37
Fruit weight (g)	254.18	82.66	482.22	99.70	21.12
Fruit length (cm)	7.23	5.05	9.22	1.11	7.70
Fruit diameter (cm)	7.59	5.31	9.52	1.08	7.83
Fruit volume	199.40	57.85	343.97	78.18	23.34
Crown length (mm)	14.02	10.31	17.64	1.96	12.73
Aril %	58.53	32.06	65.76	6.51	6.77
Fruit juiciness (%)	38.92	17.06	47.83	5.66	9.82
Number of arils/fruit	406.05	155.83	661.7	144.00	19.41
100 arils weight (g)	36.89	19.39	46.55	7.10	9.05
Rind thickness (mm)	2.98	2.1	4.36	0.50	14.03
Aril length (mm)	10.60	8.54	11.81	0.81	4.41
Aril width (mm)	7.12	6.1	7.92	0.46	6.73
Total soluble solids (°Brix)	16.90	15.09	19.34	1.10	3.07
Acidity (%)	1.36	0.3	3.32	0.97	18.66
100 seed weight (g)	2.09	1.22	2.71	0.51	6.89
Seed length (mm)	6.32	5.6	7.14	0.35	5.19
Seed width (mm)	2.57	2.08	3.13	0.24	8.18
Fruit color (L*)	64.46	27.75	80.45	10.21	9.98
Fruit color (a*)	22.71	6.2	42.22	10.13	22.69
Fruit color (b*)	33.24	18.8	43.2	6.40	13.65
Aril color (L*)	45.74	19.13	57.94	8.72	8.36
Aril color (a*)	17.00	5.93	31.43	7.00	19.55
Aril color (b*)	18.55	11.23	23.68	2.60	11.19
Aril firmness (N)	5.99	4.07	8.61	0.96	17.95
Seed texture (N)	68.07	31.01	98.31	20.71	9.82

**Table 2:** Grouping of pomegranate accessions based on Tocher’s clustering method applied to the pairwise Mahalanobis D^2^ distance calculated using the morphological data.

Cluster No.	No. of Accessions	Accession Name
Cluster I	14	IC-318720; IC-318723; IC-318734; IC-318762; IC-318754; IC-318779; IC-318790; IC-318705; IC-318753; IC-318728; IC-318703; IC-318724; IC-318706; IC-318707
Cluster II	11	P-16; P-26; G-137; Ganesh; P-13; KRS; P-23; Jallore seedless; Dholka; Jyoti; Muscat
Cluster III	5	1181; 1182; IC-318712; IC-318793; Acc no 1
Cluster IV	3	Co-white; Yercaud-1; IC-24685
Cluster V	3	Bhagawa; Phule Arakta; Ruby
Cluster VI	2	Gul e Shah Red; Gul e Shah Rose Pink
Cluster VII	1	IC-318749
Cluster VIII	1	Kabul Yellow

**Table 3:** Intra-(bold face) and inter-cluster (plain text) Euclidean distances between the eight clusters identified with Tocher’s clustering approach. Since clusters 7 and 8 are made of one accession, the within-cluster distance is not obtainable (-).

	Cluster 1	Cluster 2	Cluster 3	Cluster 4	Cluster 5	Cluster 6	Cluster 7	Cluster 8
Cluster 1	**51.48**	436.24	195.24	337.36	697.73	322.51	296.95	280.10
Cluster 2		**52.55**	511.87	267.57	223.89	316.56	659.13	192.80
Cluster 3			**76.66**	335.51	623.11	320.18	154.67	363.14
Cluster 4				**100.70**	416.07	267.25	431.94	217.86
Cluster 5					**85.20**	262.19	745.37	437.75
Cluster 6						**125.75**	376.27	352.11
Cluster 7							**-**	605.46
Cluster 8								**-**

**Table 4:** Descriptive statistics of the biochemical features of the 40 pomegranate accessions. SD—standard deviation; CV—coefficient of variation. TAA—total antioxidant activity; TP—total phenols; ANT—total anthocyanins; AA—ascorbic acid.

Parameter (Unit)	Mean	Min	Max	SD	CV (%)
TAA (mg/100 mL AA eq)	29.21	10.74	34.70	5.51	18.86
TP (mg/mL GA eq)	1523.80	875.00	2919.00	333.31	21.87
ANT (mg/100 mL c3g eq)	2.82	0.15	21.75	4.68	165.59
AA (mg/100 mL)	12.25	5.00	20.00	3.01	24.60

**Table 5:** Number of alleles (Na), polymorphic information content (PIC), and observed heterozygosity (Ho) at 29 polymorphic loci in pomegranate accessions.

SSR Locus	Na	PIC	Ho	SSR Locus	Na	PIC	Ho
PGKVR024	2	0.13	0.14	PGKVR088	2	0.09	0.09
PGKVR025	2	0.19	0.21	PGKVR092	2	0.13	0.14
PGKVR028	2	0.05	0.05	PGKVR093	3	0.42	0.50
PGKVR030	2	0.05	0.05	PGKVR098	3	0.34	0.41
PGKVR033	3	0.43	0.50	PGKVR111	2	0.17	0.18
PGKVR035	2	0.31	0.38	PGKVR114	2	0.19	0.22
PGKVR037	2	0.32	0.40	PGKVR121	2	0.37	0.49
PGKVR038	2	0.13	0.14	PGKVR123	2	0.13	0.14
PGKVR045	4	0.51	0.59	PGKVR128	2	0.33	0.42
PGKVR048	3	0.56	0.63	PGKVR131	3	0.10	0.10
PGKVR052	2	0.30	0.37	PGKVR133	2	0.37	0.50
PGKVR053	2	0.09	0.10	PGKVR135	2	0.19	0.22
PGKVR078	2	0.37	0.49	PGKVR136	2	0.25	0.29
PGKVR081	3	0.40	0.47	PGKVR142	2	0.23	0.26
PGKVR086	2	0.13	0.14				

**Table 6:** Pomegranate accessions under investigation.

Number	Name	Accession Number	Type	Origin
1	1181	IC-524027	Feral	Nainital, Uttarakhand
2	1182	IC-524028	Feral	Nainital, Uttarakhand
3	Acc. No. 1	IC-599594	Feral	Jammu, Jammu, and Kashmir
4	Phule Arakta	IC-565445	Commercial variety	MPKV, Rahuri, Maharashtra
5	Bhagawa	IC-565446	Commercial variety	MPKV, Rahuri, Maharashtra
6	Co-white	IC-595404	Cultivated variety	TNAU, Tamil Nadu
7	Dholka	IC-418167	Cultivated variety	Gujarat
8	G-137	IC-418166	Cultivated variety	MPKV, Rahuri, Maharashtra
9	Ganesh	IC-418154	Commercial variety	MPKV, Rahuri, Maharashtra
10	Gul-e-Shah Red	-	Foreign variety	Russia (formerly USSR)
11	Gul-e-Shah Rose Pink	IC-418171	Foreign variety	Russia (formerly USSR)
12	IC-24685	IC-24685	Indigenous cultivar	Banda, Uttar Pradesh
13	IC-318703	IC-318703	Feral	Mandi, Himachal Pradesh
14	IC-318705	IC-318705	Feral	Mandi, Himachal Pradesh
15	IC-318706	IC-318706	Feral	Mandi, Himachal Pradesh
16	IC-318707	IC-318707	Feral	Mandi, Himachal Pradesh
17	IC-318712	IC-318712	Feral	Mandi, Himachal Pradesh
18	IC-318720	IC-318720	Feral	Mandi, Himachal Pradesh
19	IC-318723	IC-318723	Feral	Mandi, Himachal Pradesh
20	IC-318724	IC-318724	Feral	Mandi, Himachal Pradesh
21	IC-318728	IC-318728	Feral	Mandi, Himachal Pradesh
22	IC-318734	IC-318734	Feral	Mandi, Himachal Pradesh
23	IC-318749	IC-318749	Feral	Shimla, Himachal Pradesh
24	IC-318753	IC-318753	Feral	Shimla, Himachal Pradesh
25	IC-318754	IC-318754	Feral	Shimla, Himachal Pradesh
26	IC-318762	IC-318762	Feral	Shimla, Himachal Pradesh
27	IC-318779	IC-318779	Feral	Solan, Himachal Pradesh
28	IC-318790	IC-318790	Feral	Solan, Himachal Pradesh
29	IC-318793	IC-318793	Feral	Solan, Himachal Pradesh
30	Jallore seedless	IC-418164	Cultivated variety	CIAH, Bikaner, Rajasthan
31	Jyoti	IC-595403	Cultivated variety	UAS, Bengaluru, Karnataka
32	Kabul Yellow	IC-418155	Foreign variety	Afghanistan
33	KRS	IC-595401	Local cultivar	Karnataka
34	Muscat	IC-418165	Foreign variety	Muscat, Oman
35	P-13	IC-595415	Cultivated variety	MPKV, Rahuri, Maharashtra
36	P-16	IC-595417	Cultivated variety	MPKV, Rahuri, Maharashtra
37	P-23	IC-418168	Cultivated variety	MPKV, Rahuri, Maharashtra
38	P-26	IC-418170	Cultivated variety	MPKV, Rahuri, Maharashtra
39	Ruby	IC-418158	Commercial variety	IIHR, Bengaluru, Karnataka
40	Yercaud-1	IC-418172	Cultivated variety	TNAU, Tamil Nadu

Abbreviations: MPKV—Mahatma Phule Krishi Vidyapeeth; TNAU—Tamil Nadu Agricultural University; CIAH—Central Institute for Arid Horticulture; UAS—University of Agricultural Sciences; IIHR—Indian Institute of Horticultural Research.

## Data Availability

The data not already included in the article and its Supplementary Material that supports the findings of this study are available on request from the corresponding author (R.A.M.).

## Supplementary Materials

The following supporting information can be downloaded at: https://www.mdpi.com/article/10.3390/plants11243518/s1, Table S1: Statistical analysis of the 35 quantitatively scored traits of the 40 pomegranate accessions. Table S2: Analysis of the three qualitatively scored traits of the 40 pomegranate accessions. Table S3: Correlation analysis of the 35 morphological traits. The table reports pairwise correlations (Pearson) below the diagonal and the *p* values above diagonal. Table S4: Statistical analysis of the four biochemical traits of the 40 pomegranate accessions. Table S5: primers employed in this study and their main features.
